# Early Detection of Hydrogen Leakage Using Fiber Optic Hydrogen Sensor Based on WO_3_-PdPt-Pt Nanocomposite Films

**DOI:** 10.3390/nano15110836

**Published:** 2025-05-30

**Authors:** Jixiang Dai, Zhangning Chen, Rundong Yang, Zhouyang Wu, Zhengan Tang, Wenbin Hu, Cheng Cheng, Xuewen Wang, Minghong Yang

**Affiliations:** 1 National Engineering Research Center for Fiber Optic Sensing Technology and Networks, Wuhan University of Technology, Wuhan 430070, China; djx409081947@163.com (J.D.); whutczn@163.com (Z.C.); yangrundong@whut.edu.cn (R.Y.); 345004@whut.edu.cn (Z.W.); 15623878378@163.com (Z.T.); wenbin_hu@whut.edu.cn (W.H.); cheng@whut.edu.cn (C.C.); 2 State Key Laboratory of Maritime Technology and Safety, Wuhan University of Technology, Wuhan 430063, China; 3 Sanya Science and Education Innovation Park, Wuhan University of Technology, Sanya 572000, China

**Keywords:** hydrogen sensor, optical fiber, WO_3_-PdPt-Pt nanocomposite film, light intensity demodulation

## Abstract

Quickly detecting hydrogen leakage is crucial to provide early warning for taking emergency measures to avoid personnel casualties and explosion accidents in hydrogen energy fields. Here, a compact optical fiber hydrogen sensing system with high sensitivity and quick response rate is proposed in this work. A laser diode (LD) and two photodetectors (PD) are employed as light source and optical signal transformation devices, respectively. This sensing system employs single-mode optical fiber deposited with WO_3_-PdPt-Pt nanocomposite film system as sensing element. Under irrigating power of 6 mW, the sensing probe exhibits an ultra-fast response to hydrogen concentrations of 4000 ppm and 10,000 ppm, with response times of 0.44 s and 0.34 s, respectively. In addition, detection limit of 3 ppm can be achieved by using this sensing system. The sensor also shows good repeatability during hydrogen exposure of 3~10,000 ppm, demonstrating its great potential application for hydrogen leakage in hydrogen energy facilities.

## 1. Introduction

Hydrogen, as a clean and efficient energy carrier, has attracted considerable attention in these years. However, hydrogen leakage can pose serious accidents due to its highly flammable and explosive nature. In recent years, hydrogen explosion accidents have raised higher requirements for the safe use of hydrogen energy. Consequently, monitoring hydrogen leakage safely and reliably has become extremely important for various hydrogen energy locations.

At present, commercial hydrogen sensors are mainly electrical hydrogen sensors, including these on Pt-coated W_18_O_49_ nanowire networks [1] and hierarchical Pd-WO_3_ nanoflowers [2]. However, these sensors are not nature safe due to the potential electric sparks. Compared to electrical hydrogen sensor, optical fiber hydrogen sensors have attracted many researchers’ interests, owing to their nature safety, anti-electromagnetic interference, long-distance monitoring capabilities, and adaptability to harsh environments. In recent years, significant progress has been made in the research of optical fiber hydrogen sensors, with various sensor types being proposed, such as evanescent fields sensors based on Pd_0.6_Au_0.4_ [3,4], Pd thin films [5], micro-reflector sensors deposited with Pd thin film [6], nano-platelet WO_3_ film [7], interference sensor combined with Pt-loaded WO_3_/SiO_2_ coating [8], surface plasmon resonance (SPR) sensor utilizing Pd thin film [9], fiber Bragg gratings (FBG) sensors employing Pd films [10,11], Pt/Ni composite films [12], Pt-loaded WO_3_-SiO_2_ coating [13], sensitive layer made of WO_3_ doped with Pt [14], and stimulated Raman spectroscopy sensor consisting of nanofiber [15]. Among these fiber optic hydrogen sensors, micro-reflector hydrogen sensor shows more obvious advantages such as simple structure, low cost and ease for massive production.

However, most fiber optic hydrogen sensors still have some limitations in terms of sensitivity, response rate, and cost of sensing system. To overcome these problems, a compact and economic fiber optic sensing system is proposed in this paper. Firstly, sensing probes were prepared by depositing nano-WO_3_-based composite film on end face of single-mode fiber. As the diameter of single-mode fiber is about 125 μm, it is easy to deposit hydrogen sensing films on tips of multiple optical fibers. Then, the sensing probe is set in a gas room filled with hydrogen concentration about 10% to generate microcracks by utilizing phase transition of Pd [10]. During this process, a laser diode and two InGaAs photodetectors with diameters of 300 μm (operating temperature −40~80 °C, wavelength range 800~1700 nm) are used as light source and optic signal transformation elements, which can avoid using laser diode (LD) light source and optical spectrometer, resulting in significant decrease in the cost of the sensing system. Surprisingly, sensing probe deposited with nanocomposite films show quick response rate during hydrogen exposure. This sensing system can display fast response rates and high sensitivity, which can provide a safe and economic sensing platform for hydrogen leakage detection in various facilities.

## 2. Experimental Setup and Sensor Fabrication

**Figure 1:**
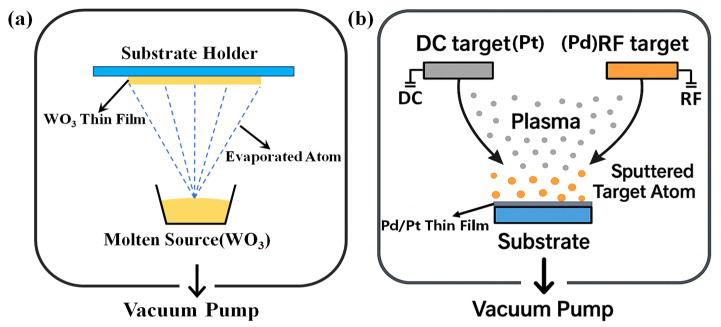
Schematic of the (**a**) evaporating and (**b**) sputtering technologies for depositing hydrogen sensitive films.

As shown in Figure 1, the preparation of sensing probes mainly involves the evaporation of WO_3_ film and the sputtering of PdPt-Pt catalytic layer. Firstly, single-mode fibers were cut by an optical fiber cleaver to form a flat tip for depositing sensitive films. Then, 210 nm WO_3_ film were evaporated on the fiber tip by using the thermal evaporation method [16,17]. During the evaporation process, oxygen was injected into chamber at a flowing rate of 200 sccm to prevent defects in the WO_3_ film. 30 nm PdPt composite films and 5 nm Pt film were sputtered on surface of WO_3_ thin films with magnetron sputtering technology. During the depositing process, the thickness of hydrogen sensitive films was monitored by quartz crystal method, and the corresponding deposition rates for WO_3_, Pd, and Pt are 0.03, 0.10, and 0.05 nm/s, respectively. Meanwhile, sensitive films are prepared on Si substrate during the depositing process for further characterization.

**Figure 2:**
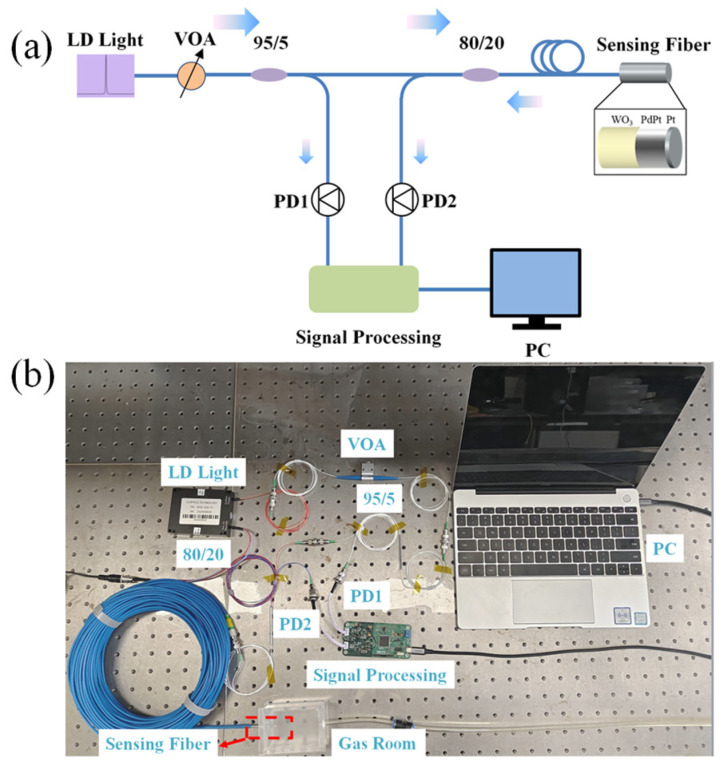
(**a**) Schematic of the optical fiber hydrogen sensor system; (**b**) a photo of the hydrogen sensing system.

As it is shown in Figure 2, an optical attenuator is used to connect the optical power of a laser diode (LD) light source and an optical coupler (95:5). The lower fraction (about 5%) power of the light source is detected by PD1, and the residual power of light source is guided to the sensing probe by an optical coupler (80:20). Electric signals of PD1 and PD2 are used to evaluate the optical power change of the LD light source and the optical power reflected by the sensing probe.

**Figure 3:**
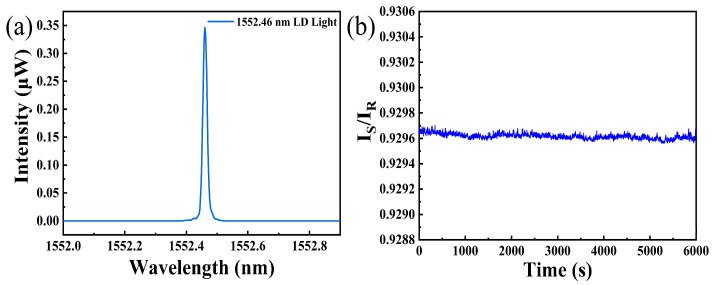
(**a**) Spectrum of the LD light source; (**b**) noise level of hydrogen sensing system with I_S_/I_R_ as a sensing parameter.

Figure 3a displays the spectrum of the LD light source. We can see that the central wavelength of the LD light source is about 1552.46 nm, which is located in low loss band for optical communication. This feature ensures long-distance detection capability of this sensing system. Figure 3b shows the fluctuations of I_S_/I_R_ about 6000 s in air. It can be observed that the fluctuation of I_S_/I_R_ is about 0.0003 during this process, and simultaneous resolution of this sensing system can be down to 0.0001. The fluctuation may be due to the variation of ambient temperature. However, utilizing I_S_/I_R_ as a sensing parameter can significantly compensate power fluctuation [17] of light source and enhance stability of the hydrogen sensing system.

Hydrogen detection tests were conducted at a room temperature of 17 °C using air as carrier gas. A rectangular acrylic box with a capacity of approximately 200 mL is used as a gas room, and there are two circular holes with a diameter about 10 mm on the opposite side walls of the gas chamber as the inlet and outlet for the mixed gas. 0.1% H_2_ (H_2_/N_2_ = 1:99, volume ratio) and 99.99% H_2_ were employed as H_2_ supplying gas, and 21% O_2_ (O_2_/N_2_ = 21:79, volume ratio) was used as dry air for hydrogen sensing. Three mass flow controllers (Beijing Sevenstar, Inc., Beijing, China, CS200A, 0~30 sccm, 0~100 sccm, 0~1000 sccm) were utilized to adjust the flowing rate of three gases, respectively. In this work, hydrogen concentrations of 3~50 ppm were provided by mixing 0.1% H_2_ with 21% O_2_ mixed gas with a total flowing rate of 1000 sccm. Hydrogen concentrations above 50 ppm were prepared by mixing 99.99% H_2_ with 21% O_2_ mixed gas at the same flowing rate. During the hydrogen testing process, all data were recorded in real-time by a computer for further analysis.

## 3. Results and Discussion

The working principle of this sensor is based on the hydrogen-induced gasochromic effect of the WO_3_-PdPt-Pt composite film, which causes the reflectivity changes of the sensing probe during hydrogen response process. There are three models [18] that are widely accepted to explain the gasochoromic mechanism of WO_3_: “double injection of ions” [19], “generation of oxygen vacancies” [20], and “localized water molecules” [21]. As sensing mechanisms between the hydrogen sensitive film and hydrogen is still controversial, the change in reflectivity of the hydrogen sensitive film is mainly attributed to the photon absorption phenomenon within the defect band in H_x_WO_3_ [19] or WO_3−x_ [20].

**Figure 4:**
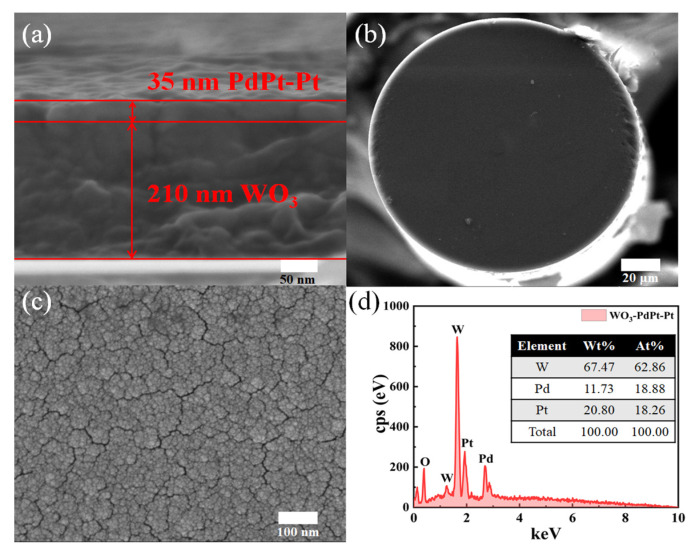
(**a**) Cross-sectional SEM image of the hydrogen sensitive film deposited on a Si substrate. (**b**) SEM image of the fiber tip. (**c**) SEM image of the sensitive film deposited on the tip of the optical fiber. (**d**) EDAX pattern of hydrogen sensitive film.

Figure 4a shows the SEM image of cross section of the hydrogen sensitive film deposited on a silicon substrate, with a thickness of 210 nm for the WO_3_ substrate layer and 35 nm for the PdPt-Pt catalytic layer. WO_3_ has excellent hydrogen induced discoloration performance [18], which can display remarkable absorption change of telecom wavelength band, resulting in obvious reflection change of optical signal in fiber core. Another advantage of using WO_3_ as basal layer is due to its good adhesion towards optical fibers. The catalytic layer consists of PdPt composite films and Pt thin film, which exhibits low volume expansion characteristics and good oxidation resistance in air environments. Stability of the hydrogen sensitive probe can be enhanced by employing a sensitive film with this membrane structure.

Figure 4b presents the SEM image of the fiber tip deposited with hydrogen sensitive film. The hydrogen sensitive film adheres firmly to the fiber tip, confirming the good mechanical stability of the sensing probe. As shown in Figure 4c, the magnified image reveals numerous microcracks with widths ranging from 5 to 10 nm. These cracks may arise from hydrogen embrittlement caused by the accumulation of Pd catalyst during hydrogen exposure [10]. However, the reflectivity of the sensing probe is not significantly affected, which may be due to the much longer operating wavelength of the LD light source. Interestingly, these microcracks can provide extra diffusion channels, which can enhance the diffusion rate of hydrogen molecules. Figure 4d gives the elemental analysis of hydrogen sensitive film. Molar ratio of W, Pd, and Pt is about 10:3:3, which is approximately consistent with the W:Pd:Pt of 210 nm WO_3_, 30 nm PdPt composite film, and 5 nm pure Pt film.

**Figure 5:**
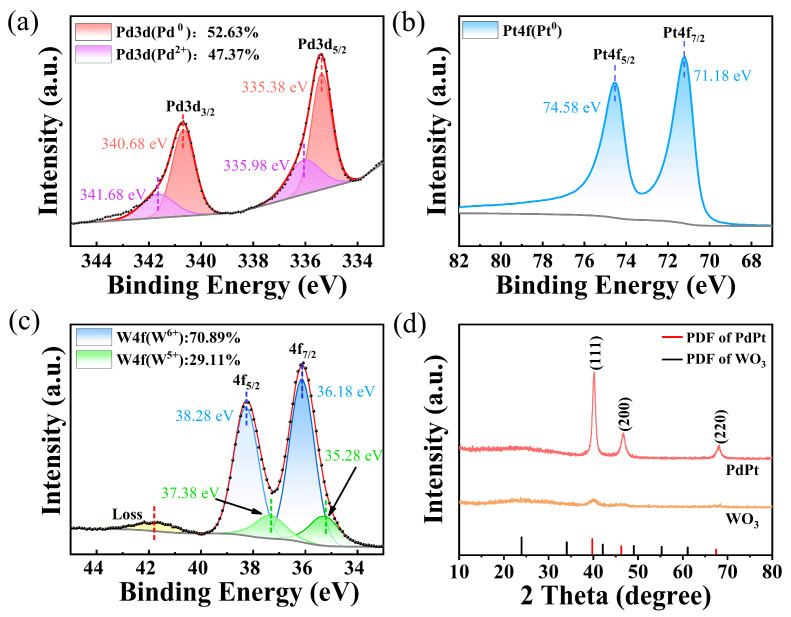
XPS of (**a**) Pd, (**b**) Pt, and (**c**) W element of WO_3_-PdPt-Pt composite films; (**d**) XRD of WO_3_-PdPt-Pt composite films.

From Figure 5a, partial Pd atoms are oxidized, which can be demonstrated by Pd 3d (Pd^2+^) spectra located at 334.46 eV and 340.06 eV. However, more than 50% of Pd atoms are Pd^0^ state. As shown in Figure 5b, the Pt 4f spectra of catalytic layer is deconvolved into Pt 4f_7/2_ and Pt 4f_5/2_ peaks, which proves their good antioxidant ability. This proves the effectiveness of the membrane structure design. The W element mainly coexists in the W^6+^ and W^5+^ states (Figure 5c), with more than 70% in the form of W^6+^ state. As shown in Figure 5d, the as-deposited PdPt-Pt film has relatively sharp peaks at 2θ = 40.6°, 47.8°, and 68.9°, confirming crystalline structure (JCPDS 65-6174) of the catalytic layer. However, WO_3_ mainly exists in an amorphous form, which is similar to the results we reported earlier [6]. Amorphous tungsten trioxide has a lower bandgap [18], which facilitates electron diffusion and may have a positive effect on faster response rates of hydrogen sensitive film [22].

**Figure 6:**
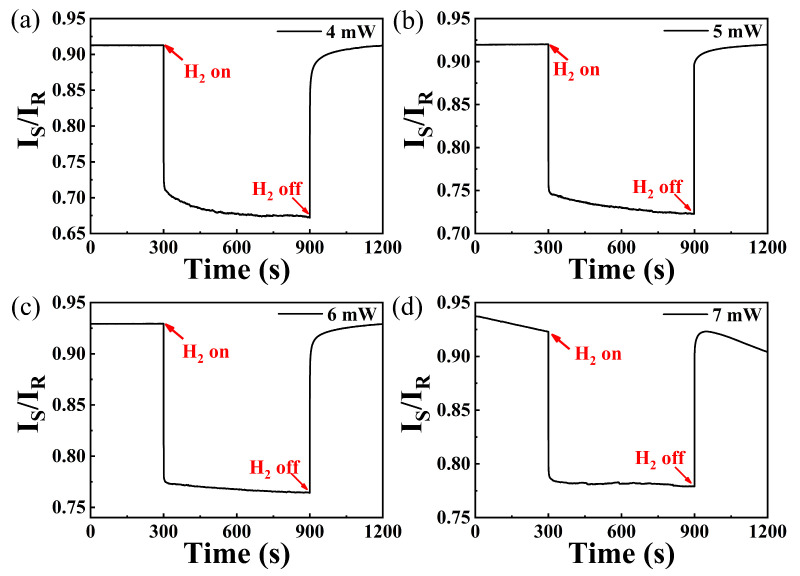
Hydrogen response of sensing probe irrigated by different optical powers: (**a**) 4 mW; (**b**) 5 mW; (**c**) 6 mW; (**d**) 7 mW.

To investigate the sensing performance of the WO_3_-PdPt-Pt composite film at different irrigating power, an optical attenuator was used to adjust the optical power reaching the fiber tip. Since primary warning value of hydrogen leakage is 4000 ppm in many scenarios, response characteristics of the sensing probe exposed to this concentration were studied at different excitation powers. Figure 6a–d depicts the 4000 ppm response characteristics of the sensing probe irrigated at different optical powers. As shown in Figure 6, I_S_/I_R_ decreased quickly as the sensing probe is exposed to 4000 ppm hydrogen at all irrigating power; then, it gradually reaches to an equilibrium in the following 5 min. Moreover, this sensing system shows a quicker response rate when the hydrogen sensitive film is irrigated at 6 mW and 7 mW. However, the stability of the sensor deteriorates as irrigating power is set to 7 mW. The reason for this phenomenon may be due to the stripping of catalysts, which can be attributed to much higher thermal expansion coefficient of Pd alloy [23]. Overall, the sensing probe exhibits better repeatability and response rate when the sensitive layer is irrigated at 6 mW. Therefore, the following hydrogen sensing test will be carried out under this power.

**Figure 7:**
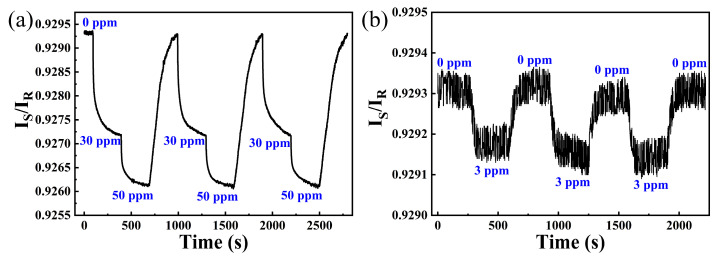
(**a**) Response of the hydrogen sensing system towards 30 ppm and 50 ppm hydrogen; (**b**) response of the hydrogen sensing system towards 3 ppm hydrogen.

As hydrogen sensing systems with better responsibility can give earlier warnings, the threshold of this sensing system was explored in this paper. Figure 7a shows three cycles of 30 ppm and 50 ppm hydrogen response curves measured by this sensing system. Although the response time for 30 ppm is more than 35 s, this sensing system still has good repeatability in the following two cycles. In addition, this sensing system can display an obvious decrease of I_S_/I_R_ during three cycles of 3 ppm hydrogen exposure (shown in Figure 7b), demonstrating its excellent ability for early warning.

**Figure 8:**
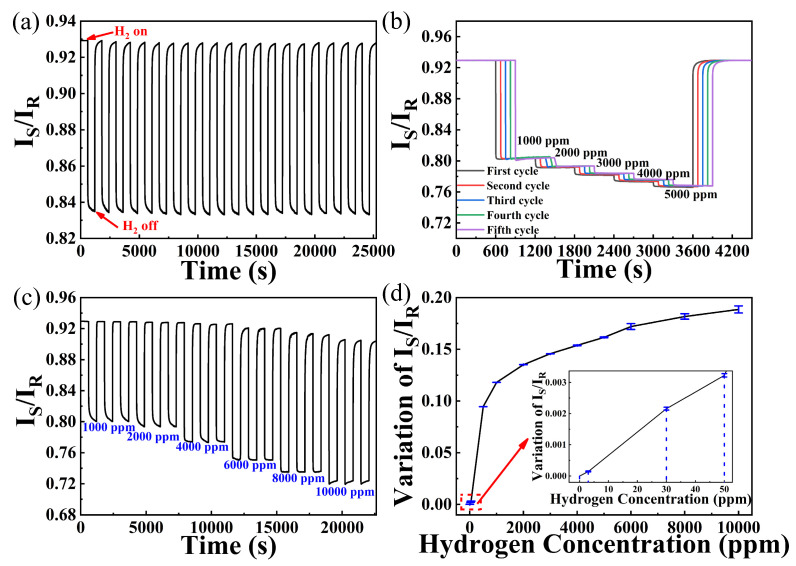
(**a**) Twenty cycles of 500 ppm hydrogen exposure; (**b**) five cycles of hydrogen sensing probe under different hydrogen concentrations; (**c**) response of different hydrogen concentrations; (**d**) decrease of I_S_/I_R_ under different hydrogen concentrations.

As displayed in Figure 8a, the hydrogen sensor exhibits about 0.95 decrease and good repeatability during 20 cycles of 500 ppm hydrogen exposure. In the following five cycles of continuous increase of hydrogen concentration exposure (shown in Figure 8b), decreases of I_S_/I_R_ for 1000, 2000, 3000, 4000, and 5000 ppm hydrogen are about 0.1268, 0.1390, 0.1495, 0.1587, and 0.1673, respectively. It can be observed in Figure 8c that the sensing probe shows faster response rates but worse repeatability with the increase in hydrogen concentration. This phenomenon is more obvious when hydrogen concentrations are above 4000 ppm. There are some hysteresis effects during the hydrogen exposure process, which gives a negative effect on hydrogen concentration monitoring. As water molecules can be generated during hydrogen reaction [21], some water molecules will be adsorbed on the surface of the sensitive film, which will hinder the reaction between the sensitive film and oxygen molecules.

From Figure 8d, we can see that this sensing system displays a nonlinear response with increase in hydrogen concentrations. This sensing system can display high sensitivity in a low concentration hydrogen atmosphere, which is beneficial for early warning of hydrogen leakage. This excellent performance indicates that the sensor can monitor hydrogen over a wide concentration range, indicating its robust capability for hydrogen detection.

**Figure 9:**
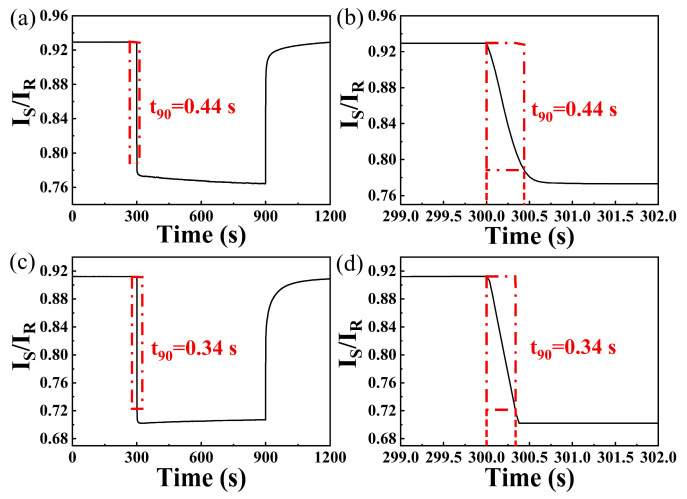
(**a**) Response curve and (**b**) response time of the hydrogen sensing probe towards 4000 ppm hydrogen; (**c**) response curve and (**d**) response time of the hydrogen sensing probe towards 10,000 ppm hydrogen.

Figure 9a,b illustrates the response curves of the sensing probe during 4000 ppm hydrogen exposure. The response time is calculated by inserting the sensing probe into the gas chamber to the sensing system reaching 90% decrease of I_S_/I_R_. And response time for 10,000 ppm hydrogen can also be observed in Figure 9c,d. The response time of this sensing system for 4000 ppm and 10,000 ppm hydrogen are about 0.44 s and 0.34 s, respectively. The quick response rate can be attributed to the joint effect of optimized irrigating power and nanocomposite thin films with multiple transmission channels. Firstly, sensitive layer with microcracks can provide more diffusion channels [2], resulting in more active sites for adsorption of hydrogen molecular. Additionally, the PdPt-Pt layer displays outstanding catalytic properties [24], which can greatly accelerate and efficiently decompose hydrogen molecules into hydrogen atoms. Moreover, hydrogen atoms diffuse faster in nanocomposite films at higher irrigating power [17], which can further accelerate the gasochromic reaction. Nevertheless, cost of this sensing system is much lower than that of previous reported work [16,25], which is an obvious merit for hydrogen sensing.

**Figure 10:**
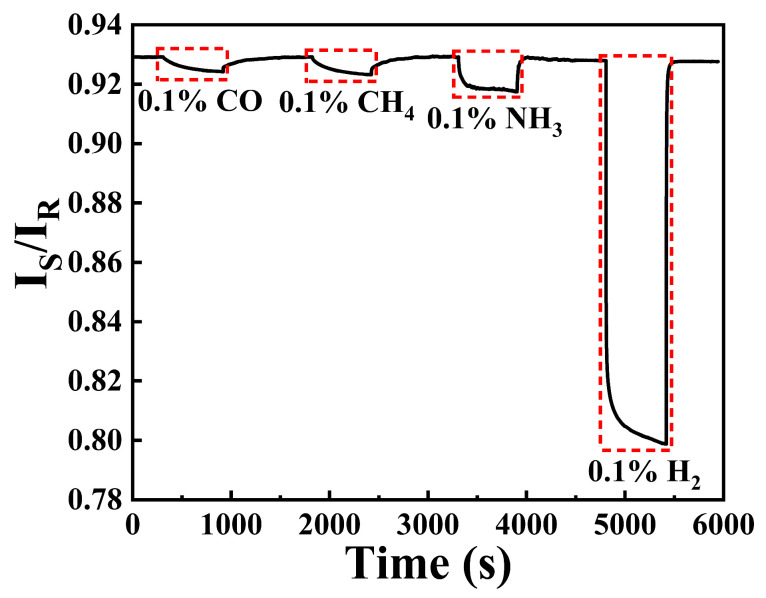
Response curve of the hydrogen sensing probe towards different gases.

To explore the selectivity of this sensing system, different gases are mixed with air and injected into gas chamber by utilizing 0.5% CO/Ar, 99.99% CH_4_, and 0.1% NH_3_/air with total flowing rate of 1000 sccm. Figure 10 depicts the response of hydrogen sensing probe towards CO, CH_4_, NH_3_, and H_2_, respectively. It can be seen that the sensing probe displays obvious response during the exposure of 0.1% CO, 0.1% CH_4_, and NH_3_. Unfortunately, the selectivity of this sensing system is worse than our previous work [16]. The reason for this phenomenon can be attributed to the increase of Pt element in the catalytic layer and higher irrigating power, as complete combustion reaction of CO and CH_4_ can be achieved with Pt as catalyst in air [26]. Then, CO_2_ and H_2_O (in air) may react to form carbonic acid, which can cause double injection of H^+^ and e^−^ in WO_3_ layer, leading to the increase in absorption of the optical single. The more obvious response of 0.1% NH_3_ may be due to the hydrogen bond between NH^4+^ and WO_3_ [27], which could also be demonstrated by the faster response rate of NH_3_ exposure.

After the exposure of CO, CH_4_, and NH_3_, the sensing probe still shows remarkable response (more than 10 times) towards 0.1% H_2_, demonstrating its better selectivity towards H_2_. However, the selectivity of this sensing system may be improved by optimizing the constitute catalytic layer and irrigating power. For oil and gas industry, utilizing hydrogen sensors based on Pt alloys [12] or Raman spectroscopy [15] maybe be more feasible, as these sensors can show repetitive response towards hydrogen in an aerobic environment.

Table 1 shows the performance comparison of several recently reported hydrogen sensors. From Table 1, we can see that the sensing system based on the WO_3_-PdPt-Pt composite film can display quicker response rate capability when compared to that of the recently reported work [25,28,29,30,31,32,33,34]. Although the detection limit of this sensing system is higher than that of recently organic hydrogen sensor [34], it can detect hydrogen far below the lower explosive limit (4%) of hydrogen in air. These merits can provide economic and reliable hydrogen leakage detecting technology for the hydrogen energy field. Furthermore, this paper can provide a clue for developing a gas sensor with excellent responsibility.

## 4. Conclusions

This work presents a novel optical fiber hydrogen sensing system based on a WO_3_-PdPt-Pt nanocomposite film. The system features a simple structure and relatively low cost. At an optimal irrigating power of 6 mW, the sensor shows ultra-fast responses to hydrogen concentrations of 4000 ppm and 10,000 ppm, with response times of 0.44 s and 0.34 s, respectively. The sensor also exhibits good repeatability during hydrogen exposure of 1000 to 10,000 ppm. Moreover, it achieves a remarkably low detection limit of 3 ppm. With its simple and compact design, the sensor can detect hydrogen concentrations far below the hydrogen explosion limit, demonstrating an economic technology for early warning of hydrogen leakage. This compact hydrogen sensing system shows advantages such as high sensitivity, rapid response speed, good repeatability, and wide monitoring range, making it promising for detecting hydrogen leakage in hydrogen energy facilities.

## Figures and Tables

**Table 1:** Comparison of reported hydrogen sensors.

Publication Year, Reference	Sensing Signal, Detection Limit, Response Time, Operating Temperature	Cost of Sensing System
2023, [2]	Electric signal, not mentioned, 1 s (500 ppm), 150 °C	Low
2012, [7]	Reflected optic spetrum, 600 ppm, less than 30 s, 100 °C	High
2018, [8]	Optical interference spectrum, about 12.5 ppm, 23 s, room temperature	High
2019, [9]	Raman spectroscopy, several ppm, less than 10 s, room temperature	High
2020, [13]	Polymer FBG spectrum, 0–0.2%, 5 ppm, tens of seconds, room temperature	High
2021, [16]	Optical intensity, 5 ppm, more than 5 s (0.4% H_2_), room temperature	Moderate
2022, [25]	Optical spectrum, 0.5 ppm, 0.9 s (0.4% H_2_), 80 °C	High
2023, [28]	Optical spectrum of FBG, 3000 ppm, less than 90 s, room temperature	High
2024, [29]	Optical spectrum of TFBG, 100 ppm, not mentioned, room temperature	High
2024, [30]	Optical transmission measurement, 1000 ppm, 25 s (4% H_2_), room temperature	High
2024, [31]	Optical spectrum of FBG, 30 ppm, 25 s (2% H_2_), room temperature	High
2024, [32]	FBG spectrum, not mentioned, tens of seconds (0. 5% H_2_), room temperature	High
2024, [33]	Frequency shift of optomechanical resonator, 710 ppm, 85 s (4% H_2_), room temperature	High
2025, [34]	Electric signal, 192 ppb, 0.84 s (1% H_2_), room temperature	Low
This work	Optical intensity, 3 ppm, 0.44 s (0.4% H_2_), room temperature	Low

## Data Availability

The original contributions presented in this study are included in the article. Further inquiries can be directed to the corresponding authors.

## References

[1] Zhu L., She J., Luo J., Deng S., Chen J., Ji X., Xu N. (2011). Self-heated hydrogen gas sensors based on Pt-coated W_18_O_49_ nanowire networks with high sensitivity, good selectivity and low power consumption. Sens. Actuators B Chem..

[2] Wang X., Meng X., Gao W. (2023). Ultrahigh-response sensor based on hierarchical Pd-WO_3_ nanoflowers for rapid hydrogen detection. Sens. Actuators B Chem..

[3] Zhao Z., Carpenter M., Xia H., Welch D. (2006). All-optical hydrogen sensor based on a high alloy content palladium thin film. Sens. Actuators B Chem..

[4] Zhao Z., Knight M., Kumar S., Eisenbraun E., Carpenter M. (2008). Humidity effects on Pd/Au-based all-optical hydrogen sensors. Sens. Actuators B Chem..

[5] Tabib-Azar M., Sutapun B., Petrick R., Kazemi A. (1999). Highly sensitive hydrogen sensors using palladium coated fiber optics with exposed cores and evanescent field interactions. Sens. Actuators B Chem..

[6] Butler M. (1994). Micromirror optical-fiber hydrogen sensor. Sens. Actuators B Chem..

[7] Ou J., Yaacob M., Campbell J., Breedon M., Kalantar-zadeh K., Wlodarski W. (2012). H_2_ sensing performance of optical fiber coated with nano-platelet WO_3_ film. Sens. Actuators B Chem..

[8] Li Y., Shen W., Zhao C., Xu B., Wang D., Yang M. (2018). Optical hydrogen sensor based on PDMS-formed double-C cavities with embedded Pt-loaded WO_3_-SiO_2_. Sens. Actuators B Chem..

[9] Lin K., Lu Y., Chen J., Zheng R., Wang P., Ming H. (2008). Surface plasmon resonance hydrogen sensor based on metallic grating with high sensitivity. Opt. Express.

[10] Sutapun B., Tabib-Azar M., Kazemi A. (1999). Pd-coated elastooptic fiber optic Bragg grating sensors for multiplexed hydrogen sensing. Sens. Actuators B Chem..

[11] Ma G., Li C., Luo Y., Mu R., Wang L. (2012). High sensitive and reliable fiber Bragg grating hydrogen sensor for fault detection of power transformer. Sens. Actuators B Chem..

[12] Kilinc N., Sanduvac S., Erkovan M. (2022). Platinum-Nickel alloy thin films for low concentration hydrogen sensor application. J. Alloys Compd..

[13] Kefer S., Dai J., Yang M., Schmauss B., Hellmann R. (2020). Hypersensitive H_2_ sensor based on polymer planar Bragg gratings coated with Pt-loaded WO_3_-SiO_2_. Opt. Lett..

[14] Caucheteur C., Debliquy M., Lahem D., Mégret P. (2008). Hybrid fiber gratings coated with a catalytic sensitive layer for hydrogen sensing in air. Opt. Express.

[15] Qi Y., Zhao Y., Bao H., Jin W., Ho H. (2019). Nanofiber enhanced stimulated Raman spectroscopy for ultra-fast, ultra-sensitive hydrogen detection with ultra-wide dynamic range. Optica.

[16] Dai J., Peng W., Wang G., Xiang F., Qin Y., Wang M., Yang M. (2017). Improved performance of fiber optic hydrogen sensor based on WO_3_-Pd_2_Pt-Pt composite film and self-referenced demodulation method. Sens. Actuators B Chem..

[17] Dai J., Li Y., Ruan H., Ye Z., Chai N., Wang X., Qiu S., Bai W., Yang M. (2021). Fiber optical hydrogen sensor based on WO_3_-Pd_2_Pt-Pt nanocomposite films. Nanomaterials.

[18] Gao C., Guo X., Nie L., Wu X., Peng L., Chen J. (2023). A review on WO_3_ gasochromic film: Mechanism, preparation and properties. Int. J. Hydrogen Energy.

[19] Lee S., Cheong H., Liu P., Smith D., Tracy C., Mascarenhas A., Pitts J., Deb S. (2001). Raman spectroscopic studies of gasochromic a-WO_3_ thin films. Electrochim. Acta.

[20] Wittwer V., Datz M., Ell J., Georg A., Graf W., Walze G. (2004). Gasochromic windows. Sol. Energy Mater. Sol. Cells.

[21] Luo J., Deng S., Tao Y., Zhao F., Zhu L., Gong L., Chen J., Xu N. (2009). Evidence of localized water molecule and their role in the gasochromic effect of WO_3_ nanowire films. J. Phys. Chem..

[22] Zhao H., Yu X., Yu S., Yang H., Guo W., Li S., Zheng J. (2025). Review on amorphous WO_3_ for eletrochromic devices: Structure, optimization strategies and applications. Mater. Today Chem..

[23] Ievlev V., Dontsov A., Kannykin S., Prizhimov A., Solntsev K., Roshan N., Gorbunov S. (2020). Thermal expansion coefficient of a Pd-Cu solid solution. Inorg. Mater..

[24] Lebon A., García-Fuente A., Vega A., Aguilera-Granja F. (2012). Hydrogen interaction in Pd-Pt alloy nanoparticles. J. Phys. Chem. C.

[25] Dai J., Ruan H., Zhou Y., Yin K., Hu X., Ye Z., Wang X., Yang M., He P., Yang H. (2022). Ultra-high sensitive fiber optic hydrogen sensor in air. J. Light. Technol..

[26] Shuk P., Mcguire C., Brosha E. (2019). Methane gas sensing technologies in combustion: Comprehensive Review. Sens. Transducers J..

[27] Zhong J., Huang B., Song J., Zhang X., Du L., Gao Y., Liu W., Kang L. (2023). Stable WO_3_ electrochromic system based on NH_4_^+^ hydrogen bond chemistry. Chem. Eng. J..

[28] Abdalwareth A., Flachenecker G., Angelmahr M., Schade W. (2023). Optical fiber evanescent hydrogen sensor based on palladium nanoparticles coated Bragg gratings. Sens. Actuators A Phys..

[29] Dissananyake K., Dewi H., Schreuders H., Bannerberg L., Abdalwareth A., Flachenecker G., Angelmahr M., Schade W. (2024). Advancing hydrogen sensing for sustainable aviation: A metal hydride coated TFBG optical fibre hydrogen sensor. e-J. Nondestruct. Test..

[30] Khanikar T., Karki D., Su Y., Hong J., Wang Y., Naeem K., Ohodnicki P. (2024). Pd/PMMA nanocomposite-coated optical fiber hydrogen sensor operating at room temperature with humidity tolerance. IEEE Sens. J..

[31] Wang C., Han Z., Wang C., Peng G., Rao Y., Gong Y. (2024). Highly sensitive fiber grating hydrogen sensor based on hydrogen-doped Pt/WO_3_. Sens. Actuators B Chem..

[32] Zhang X., Guo L., Wei X., Liu Q., Liang Y., Wang J., Peng W. (2025). Thermo-optic nanomaterial fiber hydrogen sensor. Nanomaterials.

[33] Ding W., Liu S., Chen P., Liu B., Xiao H., Ding X., Wang Y., Wang Y. (2024). Optically driven nano-beam resonator for hydrogen sensing. J. Light. Technol..

[34] Mandal S., Marsh A., Faber H., Ghoshal T., Goswami D., Tsetseris L., Heeney M., Anthopoulos T. (2025). A robust organic hydrogen sensor for distributed monitoring applications. Nat. Electron..

